# Pilot: The Holistic Concern Score. a Simple, Prescriptive and Predictive Scale for Reducing Delayed Discharge and Improving Health Outcomes

**DOI:** 10.1192/bjo.2026.11373

**Published:** 2026-06-30

**Authors:** Matilda Elliott, Benjamin Janaway, Claire Ritchie

**Affiliations:** East London NHS Foundation Trust, London, United Kingdom

## Abstract

**Aims:**

Delayed discharges arise from multiple factors and are linked with poor health outcomes, increased systemic pressures and a higher financial burden. To tackle these problems, a simple screening system (The Holistic Concern Score, HCS) was developed to identify the nature and degree of case complexity, signpost discharge barriers and enable early parallel working between allied health services.

**Methods:**

The scale was developed using pooled data over the longest-admitted patients to determine reasons for protracted stays. The two major phases of the project involved the development of the score, and subsequently a combined retrospective application to a case sample to review its efficacy and learning.

Ten factors were identified and placed into a novel scale with factors identified as either *natural* (for example treatment resistance, lack of capacity) or *systemic* (accommodation delays, funding problems). A preliminary complexity thresholdwas calculated to determine cut-off points for identifying cases for early escalation to senior management.

The same thirty cases were re-analysed approximately 3 months later to assess efficacy of the score in predicting admission duration.

**Results:**

The scale shows moderate strength in predicting length of inpatient stay and characterises discrete clusters of compounding issues signifying the need for assertive multidisciplinary management. The use of this scale at admission has led to improved holistic management of cases and provided clear therapeutic benefits, with the potential to reduce delayed discharge, improve health outcomes and relieve systemic and financial pressures.

**Conclusion:**

Further work will be needed to refine the scale but it presents promise within any inpatient service pathway as a directive, prescriptive and effective approach to case management on multiple levels.

